# Brain assembloid: a human model for neural circuits research

**DOI:** 10.1093/lifemedi/lnad031

**Published:** 2023-08-16

**Authors:** Shanshan Wu, Da Wang, Yan Liu

**Affiliations:** Institute for Stem Cell and Neural Regeneration, School of Pharmacy, Nanjing Medical University, Nanjing 211100, China; State Key Laboratory of Reproductive Medicine, Nanjing Medical University, Nanjing 211100, China; Key Laboratory of Targeted Intervention of Cardiovascular Disease, Collaborative Innovation Center for Cardiovascular Disease Translational Medicine, Nanjing Medical University, Nanjing 211100, China; Institute for Stem Cell and Neural Regeneration, School of Pharmacy, Nanjing Medical University, Nanjing 211100, China; State Key Laboratory of Reproductive Medicine, Nanjing Medical University, Nanjing 211100, China; Key Laboratory of Targeted Intervention of Cardiovascular Disease, Collaborative Innovation Center for Cardiovascular Disease Translational Medicine, Nanjing Medical University, Nanjing 211100, China; Institute for Stem Cell and Neural Regeneration, School of Pharmacy, Nanjing Medical University, Nanjing 211100, China; State Key Laboratory of Reproductive Medicine, Nanjing Medical University, Nanjing 211100, China; Key Laboratory of Targeted Intervention of Cardiovascular Disease, Collaborative Innovation Center for Cardiovascular Disease Translational Medicine, Nanjing Medical University, Nanjing 211100, China

Brain organoids that derived from human pluripotent stem cells are gradually showing their values in the study of human brain development and neurological diseases. However, human brain is composed of complex multiregional structures, and most current brain organoids are limited to recapitulate its complexity across different brain regions, especially the formation of neural circuits between brain regions. Therefore, brain assembloids emerge as required. Brain assembloids can be generated by assembling two or three brain-region organoids or integrating other lineage cells into brain organoids, thus reflecting the organ-organ or organ-cell interactions. In 2017, Birey et al. first assembled the pallium spheroids (hCS) and the subpallium spheroids (hSS) to generate forebrain assembloids, which was called fused hCS-hSS at the time [1]. Since then, the researchers named the assembly of different organoids with fused- or fusion-organoids. And until 2022, an international consortium agreed the nomenclature consensus on neural organoids and assembloids [2], which led to a final determination of “assembloids”. Based on the region-specific brain organoids technology, brain assembloids could be used to simulate more complex neurodevelopmental processes, and reflect subtler functional abnormalities in neurological diseases.

The approaches of generating brain assembloids reported by different groups is similar [1, 3]. First, two or more regions of brain organoids were differentiated separately. Then, after finishing the region-specific patterning, the regional-specific brain organoids were selected and placed into one well of ultra-low-attachment 96-well plate or a 1.5-mL Eppendorf tube, allowing them to contact with each other and spontaneously fuse to form an assembloid, which might take 1–3 days. At last, the assembloids were transferred into six-well or 60-mm ultra-low attachment plates for further culture. In short, all methods are to complete the organoids differentiation in different brain regions for the first step, and then make the two or more region-specific organoids physically close, so that they can fuse together spontaneously and form the assembloids.

The brain assembloids initially were used to study cell migration of interneuron during neurodevelopment. Birey et al. found that GABAergic neurons in ventral forebrain organoids spontaneously migrated to dorsal forebrain organoids and could functionally integrated into cortical circuits in their assembloids system, which mirrors the ventral interneuron migration during embryonic brain development [1]. In addition to interneurons migration, neuronal projection that extend axons to nearby and even distant brain regions is also a non-negligible cellular interaction in the brain. Multiple projection neurons connect different brain regions through local or long-distance synaptic connections, thereby transmitting information and performing specific functions, which is an important condition for the formation of mature neural circuits. Using the hPSCs (human pluripotent stem cells)-derived assembloids technology, researchers could deeply explore the molecular pathway and intrinsic mechanism of neural projections in the human brain. For example, Xiang et al. fused thalamic organoids with cortical organoids to construct cortico-thalamic assembloids to simulate long-range axonal connections, and found that there were mutual projections inside the assembloids, consistent with known cortico-thalamic projections *in vivo* [3]. These studies further validated the potential of brain assembloids in cell-cell interactions and circuits formation between different brain regions.

Timothy syndrome (TS) is a rare autosomal-dominant disorder associated with mutation in the CACNA1C gene. TS patients often have epilepsy, autism spectrum disorders, ventricular arrhythmias, facial dysmorphisms, immunodeficiency, and developmental delay. CACNA1C is a gene encoding the Cav1.2a subunit of the L-type calcium channel, which regulates the production of inhibitory neurons. In 2017, Birey et al. established a brain assembloid culture system derived by TS-iPSC (induced pluripotent stem cell), which has led to a better understanding of inhibitory neuronal migration patterns during brain development [1]. Real-time imaging results of assembled dorsal and ventral forebrain organoids derived from TS patients showed that the tangential-like migration of GABAergic inhibitory neurons from the ventral to the dorsal forebrain was defective, and the application of nimodipine could rescue the above phenotype by blocking the activity of L-type calcium channels. This is also the first example of using the brain assembloids approach in modeling neuronal cell interactions involving different brain regions. Using this assembloids model, the authors further explored the molecular mechanism of the disease [4]. By utilizing live cell imaging, the authors revealed that LTCC (L-type calcium channel)-mediated calcium flow could alter the length of intermediate neuronal saltations without affecting the frequency of the saltations. Whereas RNA-seq data showed that transcriptional upregulation of the GABA (γ-Aminobutyric acid) neuronal transmission pathway, ion channels, and membrane potential-related pathways could cause transcriptional upregulation of related transcriptional events dependent on LTCC activity, the authors demonstrated that GABA-A receptor function mediated saltation frequency and overall motility. The authors found that the above two distinct pathways underlie the inefficient migratory phenotype in the interneurons of TS patients. Furthermore, Andersen et al. used hiPSCs (human induced pluripotent stem cells) to reconstitute the cortico-spinal-muscle circuit, the first *in vitro* CNS (central nervous system) model of a three-unit assemboid, which could be manipulated to model motor control of muscle contraction *in vitro* [5]. The authors fused human cortical, spinal and skeletal muscle spheroids to form cortico-motor assembloids and observed spontaneous contraction of muscle cells in the assembloids. The assembloids were morphologically and functionally intact for up to 10 weeks after fusion, with more spontaneous contractions than either the muscle cells alone or the cortical-muscle assembloids. In addition, stimulation of the cortical spheroids in the assembloid with glutamate elicited a strong muscle contraction response, suggesting successful assembly of the functional cortico-spinal-muscle unit. This model could be used to characterize the developmental abnormalities and dysfunction of cortico-motor circuit, providing new tools for resolving the pathogenesis and treatment of related diseases such as amyotrophic lateral sclerosis.

**Figure 1.**
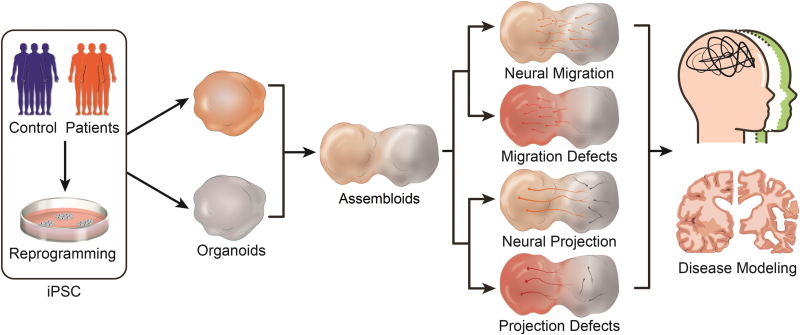
**Graphical summary of major applications of brain assembloids.** Brain assembloids are assembled with two or more region-specific organoids, and mainly are used to study neural migration, projection, and neural diseases.

Although the human brain assembloids are still in their early stages of development and continue to advance, the challenges and applications of brain assembloids are equally compelling (Fig. 1). Combined with new experimental methods, human brain assembloids technologies are expected to further explore more complex physiological and pathological phenotypes. In particular, the transformation of specific neurons into mature neural circuits, such as the development of complex cell morphologies, synaptic plasticity, and even more complex neural circuit assemblies. Through retrograde tracing and optogenetic labeling techniques, it may be one of the focuses of future research to reproduce more precise neural circuits with brain assembloids. On the other hand, the current research on human brain organoids is mostly single-brain-region-specific organoids, in which the enriched cell types are relatively simple, lacking the supplement of other different neural cell lineages, and its limitations are inevitable. However, by combining three or more different brain region-specific organoids or fusing certain brain-region organoids with more other lineage cells to construct different brain assembloids, we can further improve the *in vitro* assembly model that is closer to the bona fide human brain. In addition, brain assembloids can provide in-depth analysis of neurological diseases, especially neural circuit disorders. Combining gene editing or human-mouse chimeric brain technology, researchers can accurately interpret brain-region interactions and circuit occurrences in disease states, providing new insights and targets for future investigation and treatments.

It is still difficult to replicate the interaction between cells/tissues in human brain with brain assembloids and it still needs more exploration, however, the development of brain assembloids technology has greatly helped people understand the development of the human brain and the pathophysiology of neurological diseases. Nevertheless, there are still some open questions about brain assembloids to better study neural circuits. Firstly, construction of larger-scale assembloids requires bona fide functional vascularization which could transport oxygen and nutrients, otherwise unlimited assembly of organoids will lead to increased volume of the organoids and eventually necrosis due to central hypoxia. Secondly, the neural projections in differentiated brain assembloids are often immature, limiting their ability to mimic mature neural events such as ion channels and mature spontaneous action potentials. Lastly, current brain assembloids lack the immune system, which could be accomplished by assembling microglia and astrocytes into region-specific brain organoids.
